# In silico prediction of novel residues involved in amyloid primary nucleation of human I56T and D67H lysozyme

**DOI:** 10.1186/s12900-018-0088-1

**Published:** 2018-07-20

**Authors:** Jeddidiah W. D. Griffin, Patrick C. Bradshaw

**Affiliations:** 1 Department of Biomedical Sciences, Quillen College of Medicine, East Tennessee State University, Johnson City, TN 37614 USA; 2 Department of Biomedical Sciences, Quillen College of Medicine, East Tennessee State University, Johnson City, TN 37614 USA

**Keywords:** Amyloidosis, Lysozyme, Residues interaction networks, Native structure, *B*-factor

## Abstract

**Background:**

Amyloidogenic proteins are most often associated with neurodegenerative diseases such as Alzheimer’s disease, Parkinson’s disease, and Huntington’s disease, but there are more than two dozen human proteins known to form amyloid fibrils associated with disease. Lysozyme is an antimicrobial protein that is used as a general model to study amyloid fibril formation. Studies aimed at elucidating the process of amyloid formation of lysozyme tend to focus on partial unfolding of the native state due to the relative instability of mutant amyloidogenic variants. While this is well supported, the data presented here suggest the native structure of the variants may also play a role in primary nucleation.

**Results:**

Three-dimensional structural analysis identified lysozyme residues 21, 62, 104, and 122 as displaced in both amyloidogenic variants compared to wild type lysozyme. Residue interaction network (RIN) analysis found greater clustering of residues 112–117 in amyloidogenic variants of lysozyme compared to wild type. An analysis of the most energetically favored predicted dimers and trimers provided further evidence for a role for residues 21, 62, 104, 122, and 112–117 in amyloid formation.

**Conclusions:**

This study used lysozyme as a model to demonstrate the utility of combining 3D structural analysis with RIN analysis for studying the general process of amyloidogenesis. Results indicated that binding of two or more amyloidogenic lysozyme mutants may be involved in amyloid nucleation by placing key residues (21, 62, 104, 122, and 112–117) in proximity before partial unfolding occurs. Identifying residues in the native state that may be involved in amyloid formation could provide novel drug targets to prevent a range of amyloidoses.

## Background

Amyloidoses are a group of diseases defined by the formation of protein aggregates characterized by stacks of cross-beta sheets [1]. There are dozens of different amyloid diseases caused by a variety of both wild type (WT) and mutant forms of proteins [2]. Some of the most well-known amyloidoses are neurodegenerative diseases such as Alzheimer’s disease (involving amyloid-beta peptide) and Parkinson’s disease (involving alpha-synuclein protein). However, not all amyloid diseases affect the brain. Lysozyme amyloidosis is a rare disease characterized by the deposition of amyloid fibrils of the enzyme lysozyme. Lysozyme was discovered by Alexander Fleming in 1922 [3] and is an antimicrobial enzyme synthesized by hepatocytes, cells of the gastrointestinal system, and macrophages [4]. Lysozyme amyloidosis has no known effective treatment and leads to lysozyme amyloid deposits typically concentrated in the liver [5], spleen, gastrointestinal tract [6], and kidneys [7]. Lysozyme amyloidosis is thought to be largely caused by subtle structural changes of the protein caused by genetic mutations that lead to pockets of local instability and a greater likelihood of partial unfolding [8]. The Online Mendelian Inheritance in Man (OMIM) database [9] entry for lysozyme (OMIM ID 153450) reports four lysozyme variants that are associated with the disease: I56T [10], D67H [10], W64R [11], and F57I [12].

Lysozyme has long been used as a model for studying protein structure and folding. Since lysozyme is structurally and functionally well-characterized, the protein provides a useful model for understanding the complex process of amyloid fibril formation [13]. Several studies have investigated the role of amyloidogenic mutations on lysozyme amyloid formation with a focus on the first identified mutations, I56T and D67H. Studies that examined the crystal structure of the WT and I56T variant suggest very little difference in the native structure of these enzymes [14]. The D67H variant, however, destroys the hydrogen bonds that stabilize the beta-domain, leading to the displacement of a long loop of residues [15]. Because the obvious loop displacement between the WT and D67H mutant is not present in the I56T mutant, it is thought that this change is not responsible for amyloidogenesis. Instability of the I56T variant may be caused by subtle changes in bonding between alpha and beta domains of lysozyme; similar bonding changes are also evident in the D67H variant [15].

Since the structure of proteins in amyloid plaques are different from the native structure, amyloidogenic proteins must at least partially unfold during amyloidogenesis. Most studies focus on the unfolding process of lysozyme instead of differences in the native structure. The amyloidogenic proteins likely spend more time partially unfolded, providing more opportunities for unfolded segments to interact and aggregate in the form of amyloid plaques [8]. The importance of the partially unfolded state for lysozyme amyloidosis has been demonstrated in vitro with the use of antibodies that stabilize the protein [16, 17]. Studies have also shown that both I56T and D67H are less stable than WT lysozyme when heated [15], and I56T is also less stable than WT at low pH [14, 15, 18, 19], further supporting a role for instability. However, other factors besides regions of protein instability may be involved in amyloidogenesis. Further studies that examine primary nucleation from different perspectives could provide more insight into this important process that is associated with a variety of diseases.

Residue interaction networks (RIN) abstract protein structure into a network of likely side-chain interactions with residues represented as nodes and interactions represented as edges, the connections between the nodes [20]. Several metrics are available for studying networks and identifying subnetworks of interest [21–23]. Many network features have been associated with and applied to protein structural and functional characteristics [22, 24–26], demonstrating the relevance of RINs to structural biology. Clusters are particularly interesting in RIN analysis because they identify areas with many chemical interactions, suggesting structural rigidity or functional importance [27]. RIN analysis is most useful when combined with 3D structural analysis [28]. This study uses the two-pronged approach of combining 3D structural analysis with RIN analysis to identify residues in the native structure that are likely involved in amyloid formation.

## Methods

### Three-dimensional structure visualization and structure comparison

The Online Mendelian Inheritance in Man (OMIM) database [9] was searched for mutations in lysozyme that have been associated with amyloidosis. The Protein Data Bank (PDB) [29] was then searched for human lysozyme structures with these mutations, resulting in a dataset of WT human lysozyme (PDB ID: 1REX, [30]) and two amyloidogenic variants, I56T (PDB ID: 1LOZ, [15]) and D67H (PDB ID: 1LYY, [15]). The 3D protein structure coordinates were downloaded from the PDB and visualized using UCSF Chimera v1.11.2 software [31]. The 3D structures were overlapped using the MatchMaker application [32] in UCSF Chimera with default settings, and the root mean square deviations (RMSDs) for the full residues from the wild type (1REX) structure of lysozyme were calculated in the Multialign Viewer [32]. Side chains that had different locations when compared to WT lysozyme in both amyloidogenic variants were selected for further study. Because the resolution of the PDB files used for the comparison was less than or equal to 1.8 Å, only residues with a RMSD from WT greater than or equal to 1.9 Å were considered.

### Generating residue interaction networks and calculating clusters and metrics

To detect network clusters in the proteins, the PDB files were converted to GML format using the Protein Graph Converter software from the Protein Graph Repository (PGR) [33]. Each alpha carbon was considered a node, and an edge was drawn between every alpha carbon within seven angstroms of another. The GML files were then analyzed for clusters using the MCODE application in the network analysis software Cytoscape v3.4.0 [34]. Only clusters with MCODE scores greater than or equal to 5.00 were selected for further analysis. The residues involved in the clusters in the amyloidogenic mutants were compared to those identified in WT lysozyme. As with the 3D structure comparison, cluster changes that are in common between the amyloidogenic mutants and different from WT lysozyme were selected for further analysis. UCSF Chimera was used to calculate the average residue *B*-factor for each of the clusters. The Pearson correlation of MCODE scores for the selected clusters of each of the lysozyme PDB files with the average *B*-factor for the clusters was calculated using GraphPad Prism 7, and a *p*-value of < 0.05 was considered significant.

### Generating predicted dimer and trimer structures and calculating interprotein bond number and energies

ClusPro v2.0 software [35] was used to generate predicted structures of homodimers, homotrimers, and heterodimers for WT lysozyme and the amyloidogenic variants. After multimer generation, the resulting PDB files for the top predicted dimer and trimer structures were edited so that each lysozyme protein was given a unique name. Next, the edited PDB files were uploaded to the Residue Interaction Network Generator (RING) v2.0 software [36], and residue interaction networks were created using a strict distance threshold between the closest atoms of residues separated by at least two other residues. Multiple edges per residue pair were allowed but only one edge per interaction type. The resulting graph files were analyzed for the number of interactions and overall bonding energy occurring between lysozyme proteins in dimers or trimers. The number and bond energies of the interprotein interactions were analyzed for the whole complexes and the residues of interest to provide information about the relative importance of the residues of interest to the formation of dimers and trimers.

## Results

### Three-dimensional structural comparison of lysozyme

**Fig. 1:**
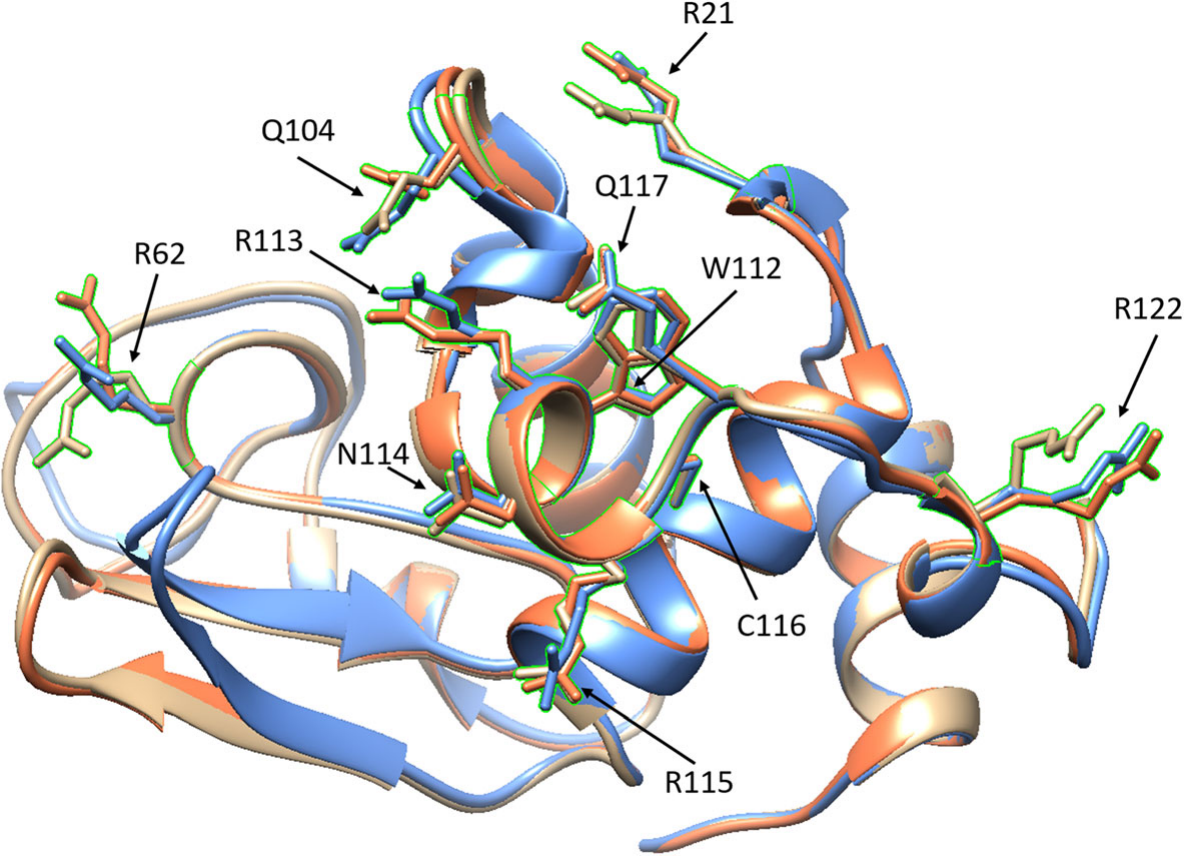
Three-dimensional overlap of lysozyme structures 1REX (WT, tan), 1LOZ (I56T, orange), and 1LYY (D67H, blue). The side chains of residues 21, 62, 104, 112–117, and 122 are shown and outlined in green

Three-dimensional structure overlaps from the MatchMaker software revealed residues of the amyloidogenic variants that differed from WT lysozyme. The D67H variant diverges more from WT lysozyme than the I56T variant. However, there are only four residues with a RMSD greater than or equal to 1.9 Å that were shared by both the I56T and the D67H amyloidogenic lysozyme variants: residues 21, 62, 104 and 122. In all four cases, the structural changes are in proximity to each other in the 3D structure (Fig. 1).

### Residue interaction network clustering analysis of lysozyme structures

**Fig. 2:**
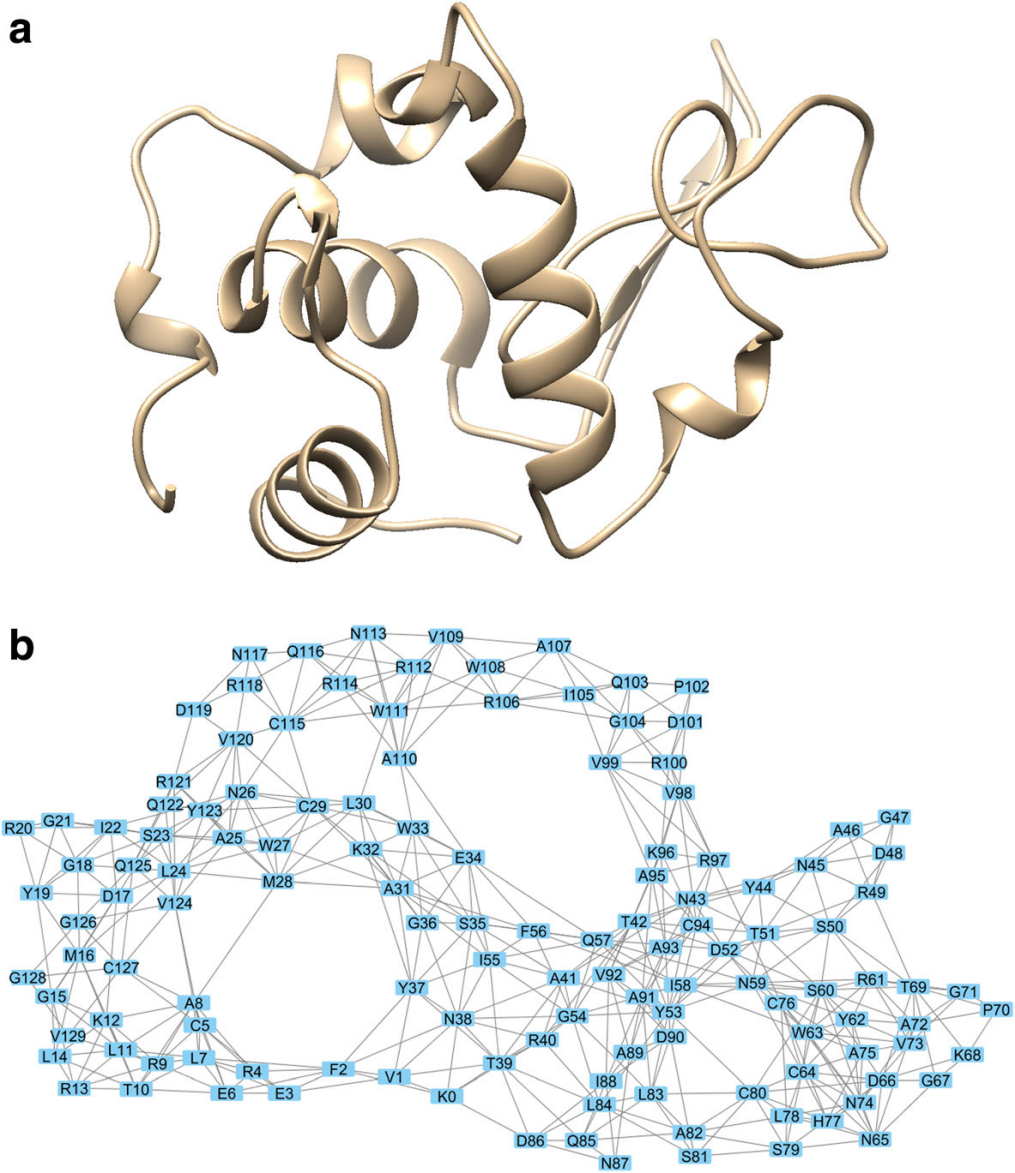
**a** Three-dimensional WT lysozyme, PDB 1REX. **b** Network representation of WT lysozyme with amino acids represented as nodes and edges drawn between alpha carbons within seven angstroms of each other. Residue numbering only for this image starts at zero

The 3D structure of lysozyme and its resulting PGR residue interaction network (RIN) representation are shown in Fig. 2. The MCODE application in Cytoscape revealed four clusters in each of the lysozyme structures that had an MCODE score of greater than 5.00. Some of the clusters in each of the lysozyme variants involved similar or identical sets of residues. As before, we focused on the differences from the WT clusters that were present in both amyloidogenic proteins. The most robust and consistent difference was the cluster around residues 112–117. The MCODE score for the cluster containing these residues in WT lysozyme was 5.11, but it increased in both amyloidogenic variants to 6.00. Residues 104 and 106–108 are clustered with 112–117 in WT lysozyme but not in I56T and D67H. The average *B*-factors of the clusters containing residues 112–117 also decreased in both amyloidogenic variants compared to WT lysozyme. These results are shown in Table 1. Residues 112–117 are shown in Fig. 1. There was a statistically significant negative correlation (r^2^ = 0.44, *p* = 0.0184) between MCODE scores for the top four clusters in each of the three lysozyme network structures and the average *B*-factor for each cluster. The residues are in proximity to the other residues of interest as shown in Fig. 1.

**Table 1:** RIN clusters in WT and amyloidogenic variants of lysozyme

Lysozyme Structure	Cluster Rank	MCODE Score	Residues Involved	Average Residue *B*-Factor
WT				14.88
	1	6.36	92–103	12.35
	2	6.00	32–37	11.71
	3	5.60	7–13, 19, 23–29, 31	9.72
	4	5.11	104, 106–108, 112–117	19.09
I56T				16.06
	1	6.00	112–117	17.53
	2	6.00	92–98	10.54
	3	5.60	7–13, 19, 23–29, 31	10.39
	4	5.00	122–126	29.15
D67H				13.24
	1	6.50	92–100	7.83
	2	6.00	112–117	14.99
	3	6.00	32–37	8.21
	4	5.75	7–13, 17–19, 23, 25–29, 31	8.12

### Interprotein bonds involving residues 21, 62, 104, 112–117, and 122 in predicted lysozyme dimers and trimers

**Fig. 3:**
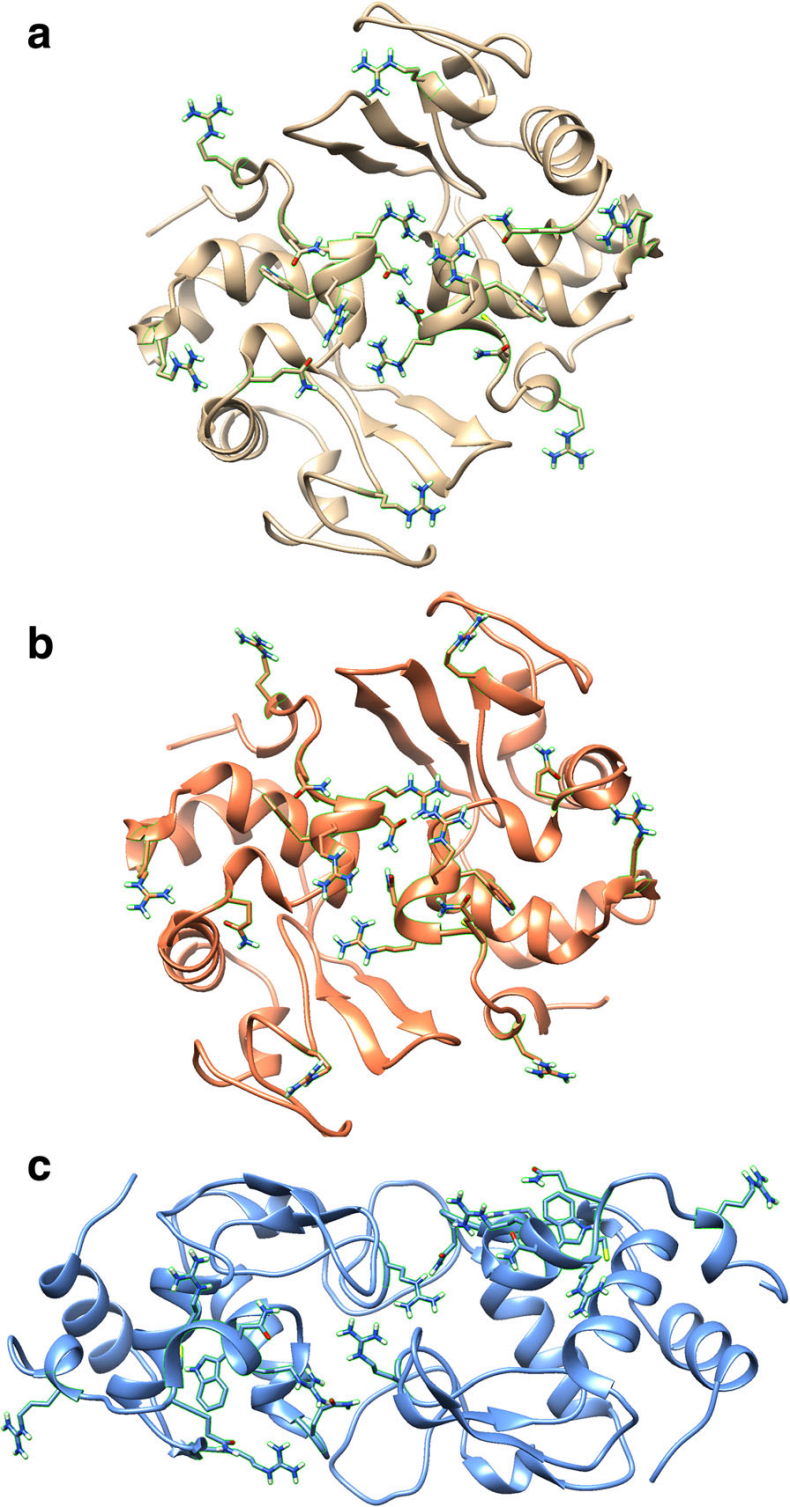
Predicted 3D structures of WT or mutant lysozyme homodimers. **a** WT: WT, (**b)** I56T: I56T, and (**c**) D67H: D67H. The side chains of residues 21, 62, 104, 112–117, and 122 are shown and outlined in green

**Fig. 4:**
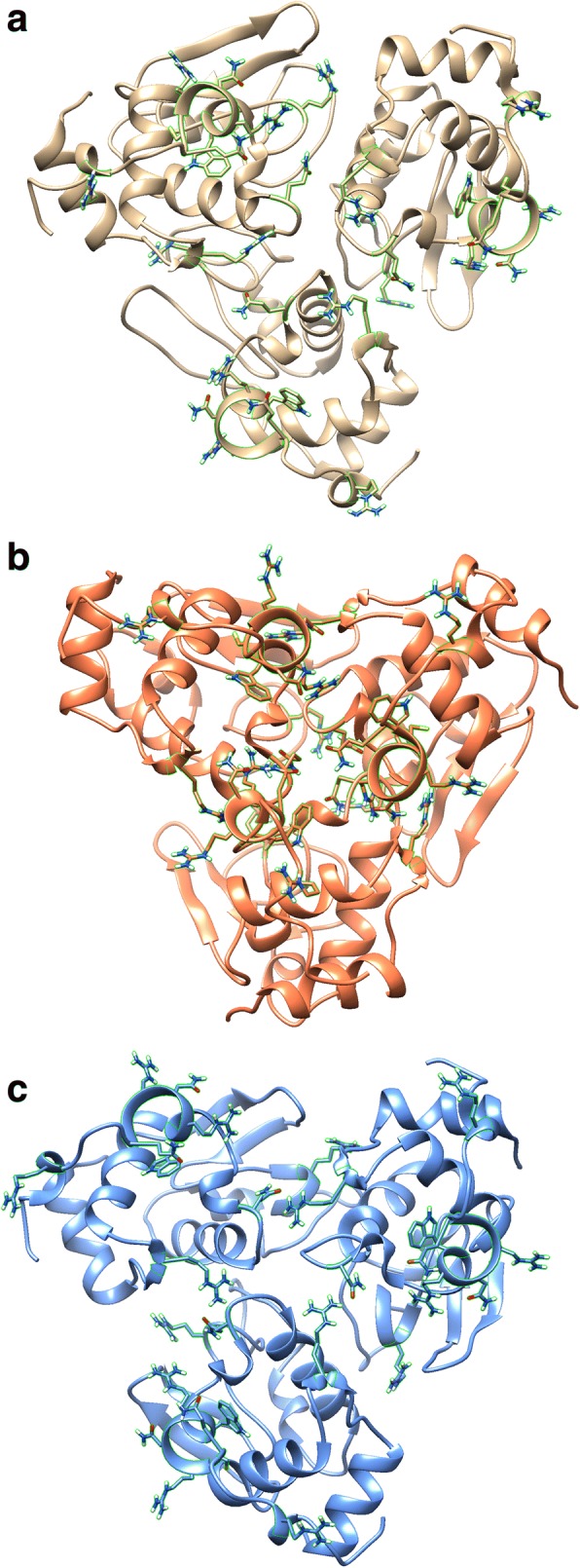
Predicted 3D structures WT or mutant lysozyme homotrimers. **a** WT: WT: WT, (**b**) I56T: I56T: I56T, and (**c**) D67H: D67H: D67H. The side chains of residues 21, 62, 104, 112–117, and 122 are shown and outlined in green

**Fig. 5:**
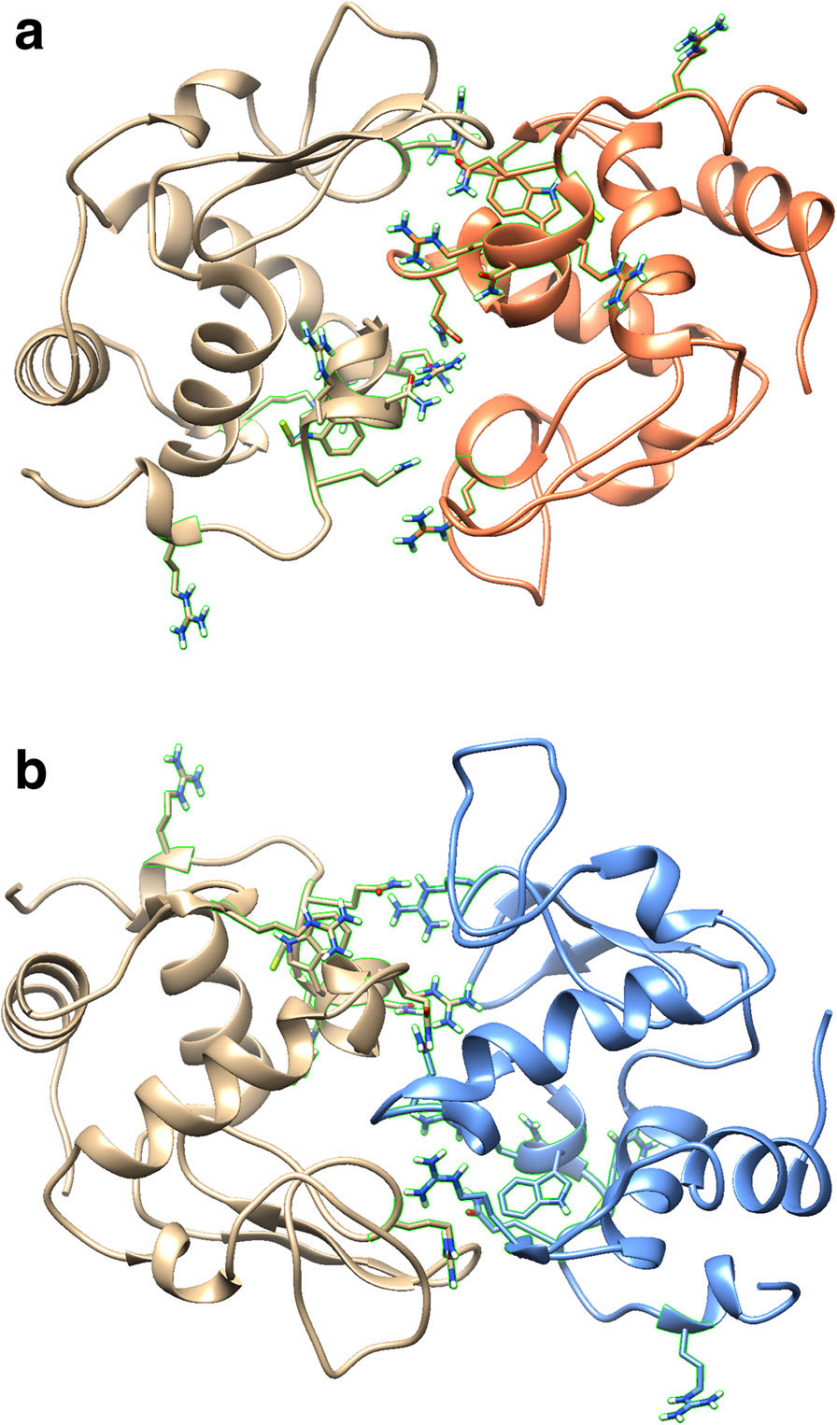
Predicted 3D structures of heterodimers of each of the amyloidogenic lysozyme variants with WT lysozyme. **a** WT: I56T, (**b**) WT: D67H. The side chains of residues 21, 62, 104, 112–117, and 122 are shown and outlined in green

The top-rated ClusPro models of dimer and trimer structures for each of the variants of lysozyme are shown in Fig. 3 (homodimers), Fig. 4 (homotrimers), and Fig. 5 (heterodimers). The number of interprotein residue interactions and the strength of the bonding energy for these interactions for all residues and for the residues of interest were quantified using RING 2.0 software, and the results are shown in Table 2 (homodimers), Table 3 (homotrimers), and Table 4 (heterodimers).

**Table 2:** ClusPro predicted interprotein binding energies for WT and mutant lysozyme homodimers

Lysozyme Structure	Number of Interprotein Residue Interactions		Interprotein Bond Energy (kJ/mol)	
	Total	Residues of Interest (% of Total)	Total	Residues of Interest (% of Total)
WT	100	36 (36%)	1019	398 (39.1%)
I56T	71	35 (49.3%)	768	442 (57.6%)
D67H	61	18 (29.5%)	594.6	246 (41.4%)

**Table 3:** ClusPro predicted interprotein binding energies for WT and mutant lysozyme homotrimers

Lysozyme Structure	Number of Interprotein Residue Interactions		Interprotein Bond Energy (kJ/mol)	
	Total	Residues of Interest (% of Total)	Total	Residues of Interest (% of Total)
WT	89	2 (2.2%)	824.8	12 (1.5%)
I56T	133	74 (55.6%)	1392.8	817 (58.7%)
D67H	108	24 (22.2%)	1060.8	320 (30.2%)

**Table 4:** ClusPro predicted interprotein binding energies for WT and mutant lysozyme heterodimers

Lysozyme Structures	Number of Interprotein Residue Interactions		Interprotein Bond Energy (kJ/mol)	
	Total	Residues of Interest (% of Total)	Total	Residues of Interest (% of Total)
WT: I56T	48	25 (52.1%)	481	238 (49.5%)
WT: D67H	49	27 (55.1%)	482.6	289.6 (60.0%)

For homodimers, residues of interest made up 36% of the number of interprotein residue interactions in WT lysozyme, contributing to 39.1% of the predicted interprotein bonding energy. The overall number of interprotein residue interactions increased to 49.3% of the total in I56T, but it decreased to 29.5% of the total number in D67H. However, the total percentage of interprotein bonding energy contributed by the residues of interest increased for both amyloidogenic variants even though the total interprotein bonding energy for dimers was less than WT dimers.

Trimers showed different trends from the homodimers (Table 3). The total number of interprotein residue interactions increased for I56T (133 interactions) and D67H (108 interactions) compared to WT (89 interactions). The contribution of the residues of interest to the total number of interprotein residue interactions increased from 2.2% for WT to 55.6% for I56T and 22.2% for D67H. Interprotein bonding energy showed similar trends. The total interprotein bonding energy increased from 824.8 kJ/mol in the WT to 1392.8 kJ/mol in I56T and 1060.8 kJ/mol in D67H. Residues of interest contributed 1.5% of the total interprotein bonding energy in the WT but 58.7% in I56T and 30.2% in D67H.

Heterodimers (WT: I56T and WT: D67H) showed fewer and less energetic interprotein interactions than homodimers (Table 4). The WT: I56T heterodimer had 48 interprotein interactions and 481 kJ/mol interprotein bonding energy, and the WT: D67H heterodimer had 49 interprotein interactions and 482.6 kJ/mol bonding energy. In both cases, there was a high reliance on the residues of interest for the bonding. The residues of interest made up more than 50% of the number of interprotein bonding interactions and nearly 50% of the interprotein bonding energies of both heterodimers.

## Discussion

### Three-dimensional structural comparison suggests residues 21, 62, 104, and 122 may be involved in lysozyme amyloidogenesis

Comparing 3D protein structures revealed that both amyloidogenic variants of lysozyme analyzed differ from WT lysozyme in the location of residues 21, 62, 104, and 122. Following the reasoning of Booth and colleagues [15], because these differences are common to both amyloidogenic variants, they suggest these residues may play a role in the formation of amyloid fibrils. The four residues (21, 62, 104, and 122) are in proximity to each other in all three variants (Fig. 1). As discussed above, the prevailing hypothesis for lysozyme amyloidosis is that the mutations disrupt the hydrogen bonds near the residues between alpha and beta domains, leading to partial unfolding followed by fibril formation [8, 19, 37]; interprotein interactions between the native structures are not thought to play a large role. However, the consistency of the native structure changes observed in both amyloidogenic variants hints at a role for the residues in the native structure in amyloidosis. We hypothesized that these residues may facilitate an interprotein interaction between native state amyloidogenic lysozyme proteins, contributing to the first steps of amyloidosis. To gather further support for this hypothesis, we examined the structures for network cluster changes.

### Residues 112–117 may also be involved in lysozyme amyloidogenesis

In addition to the 3D structural changes described above, amyloidogenic variants of lysozyme were associated with changes in network clusters. The network cluster consisting of residues 112–117 of I56T and D67H had the greatest and most consistent cluster changes, showing an increased MCODE score in both variants compared to WT (Table 1). Residues 104 and 106–108 were included in the cluster containing residues 112–117 in WT lysozyme only, so the loss of this part of the cluster may also have structural implications. More clustering has been shown to be associated with greater structural stability [27, 38], so we hypothesized that residues 112–117 have greater structural stability in the amyloidogenic variants compared to WT lysozyme. To test this hypothesis, we used UCSF Chimera to calculate the average *B*-factor for each of the residues in each of the PDB files. The *B*-factor is a measure of flexibility where a lower *B*-factor indicates greater stability [39]. Even in our relatively small data set of three lysozyme structures, we found a statistically significant negative correlation (r^2^ = 0.44, *p* = 0.0184) between MCODE scores and average *B*-factors for the top four clusters of each of the PDB files shown in Table 1. Consistent with the hypothesis of greater cluster stability, residues 112–117 had smaller average *B*-factors in amyloidogenic variants compared to the cluster containing these residues in WT lysozyme. Because most studies focus on the instability caused by amyloidogenic mutations, disrupting this cluster while stabilizing other regions may provide a novel therapeutic approach. The side chains of residues 112–117 are shown in Fig. 1 along with the other residues of interest (21, 62, 104, and 122) from 3D structural comparison. Residues 112–117 are in proximity to the residues identified through 3D structural comparison. Therefore, residues 112–117 may also be involved in facilitating interactions between different lysozyme molecules and possibly contribute to primary nucleation of amyloid fibrils. To test this hypothesis, we simulated intermolecular interactions between native state structures.

### Predicted dimer and trimer structures provide further evidence for the involvement of residues 21, 62, 104, 112–117, and 122 in lysozyme amyloidogenesis

To test the hypothesis that residues 21, 62, 104, 112–117, and 122 in the structure of amyloidogenic lysozyme variants are involved in primary nucleation, we used ClusPro docking software to predict the 3D structure of dimers and trimers of lysozyme for the PDB files. While ClusPro generates the structures of many predicted dimers and trimers, we only analyzed the top-ranked structures. Visual inspection of the predicted homodimers (Fig. 3) suggested some of the residues of interest may be involved in dimer interactions. To test this hypothesis, we examined the number and strength of interprotein residue interactions (Table 2). The number of interprotein bonds was less for amyloidogenic variant homodimers, and there was no consistent trend with the percent contribution of the residues of interest to the number of interprotein bonds in the dimers. Both amyloidogenic variant homodimers had overall less bonding energy. However, the residues of interest contributed to a greater degree to the interprotein bonding energy compared to WT lysozyme. Overall, the contribution of the residues of interest to lysozyme homodimer formation in amyloidogenic variants was not as convincing as the evidence for their role in simulated trimer formation.

Visual inspection of the predicted trimer structures of lysozyme suggested a greater role for the residues of interest in interprotein interactions of amyloidogenic variants than WT lysozyme (Fig. 4). When examined quantitatively, both amyloidogenic variants had stronger and more numerous bonds between proteins compared to WT (Table 3). This suggests the residues of interest may be largely facilitating the predicted sharp increase in interprotein interactions, supporting the important role predicted for these residues by 3D structural analysis and network analysis.

### A role for mutant native structure in enhancing lysozyme amyloid fibril formation?

Taken together, these data support the hypothesis that residues 21, 62, 104, 112–117, and 122 in the mutant native states are involved in lysozyme amyloid primary nucleation. While the instability of amyloidogenic variants of lysozyme is almost certainly the most important factor for fibril formation [40], it may not be the only factor involved. The greater interprotein interactions predicted to occur between trimeric amyloidogenic mutants compared to WT lysozyme may lead to amyloidogenic variants oligomerizing more readily before unfolding. We hypothesize these aggregates are composed of mostly mutant proteins because heterodimers with WT lysozyme have less interprotein bonding energies than mutant homodimers. This suggests when one of the lysozyme variants partially unfolds, it may already be in proximity to or bound to another mutant molecule, leading to a greater probability of amyloid nucleation. The sequence of events of the unfolding process for WT lysozyme and the I56T variant are consistent with our hypothesis. It has been demonstrated that lysozyme alpha helices A, B, and D [41] are some of the last regions to unfold [42]. All the residues identified in this report except residue 62 are in or near regions of the protein that unfold later in the process, so these positions have a greater chance of maintaining their structure to facilitate intermolecular interactions during this process.

Furthermore, Ahn and colleagues concluded that mutations in the alpha domain of lysozyme are less likely to influence the formation of transient intermediate states compared to mutations in the beta domain [43]. The resistance of changes in the alpha domain to form transient intermediates is largely consistent with our findings which suggest subtle changes in the position and interactions of residues 21, 62, 104, 112–117, and 122 (largely in the alpha domain) play a role in the early steps of primary nucleation before significant unfolding of the alpha domain occurs. Sequence-based predictions of “hot spots” of aggregation using Aggrescan [44] and TANGO [45] suggest sequences in both the alpha and beta domains may be important for amyloid formation. While we also report a role for residues in the alpha domain, the residues identified here are not all included in the results of the sequence-based predictions. This may be because we predict residues 21, 62, 104, 112–117 and 122 to play a role in amyloidogenesis prior to the formation of unfolded intermediates by positioning lysozyme mutants in or near the native state close to each other. Because this is predicted to occur before significant unfolding and fibrillization of lysozyme, it is understandable that sequence-based algorithms did not identify the same residues. Fibrillization is a multistep process, and it is likely that different residues may be involved in different steps.

Residues 21, 62, 104, and 122 do not appear to be involved in increasing the flexibility of lysozyme in the amyloidogenic mutants because the total predicted bond energies from RING analysis for monomers do not suggest consistent and structurally important differences when compared to WT. Furthermore, the average *B*-factors for these residues in mutant lysozyme do not consistently differ from WT. It has been suggested that residues not present in the partially unfolded region can be altered without affecting the process of amyloid fibril formation [46], but our data challenge this suggestion. It has been noted that lysozyme amyloid plaques are nearly entirely composed of the amyloidogenic variant free from WT protein [18]. This may be due not only to the greater instability of amyloidogenic proteins, but also due to the predicted favored intermolecular interactions of homodimers and homotrimers compared to heterodimers (Table 4). A better understanding of the process of amyloidogenesis for lysozyme could yield insights into treatments for many different types of amyloidoses.

### Study limitations and future studies

Data from various computational approaches used in this study support a role for lysozyme residues 21, 62, 104, 112–117, and 122 in lysozyme amyloidosis. However, this study has several limitations. The most obvious limitation is the small sample size of 3D structures used. Unfortunately, the study is limited by the availability of PDB files of human amyloidogenic lysozyme variants. We draw our conclusions from three PDB files, making this a preliminary study. The findings reported here may not be shared by all amyloidogenic variants of lysozyme. To increase confidence in our conclusions, further studies should explore the structures of WT and amyloidogenic lysozyme under various experimental conditions. In addition, it was found that different software programs predicted different lowest-energy oligomeric structures that influence results. Furthermore, these hypotheses need to be experimentally tested to verify the importance of these residues for amyloidogenesis. Lysozyme mutants with smaller side chains or nonpolar side chains at the residues of interest could be created and the fibril formation kinetics studied. Data from studies of double mutants may also be useful. Future studies could be performed where residues 21, 62, 104, 112–117, or 122 are mutated in combination with I56T or D67H and tested for altered fibril formation kinetics. However, it may be most useful to mutate residue 21 and a residue in 112–117 or 122 because residues 32 through 108 have been shown to form the core of the lysozyme fibril [47]. Therefore, mutating these residues may interfere with amyloid formation after unfolding.

## Conclusions and testable hypotheses generated

This preliminary study used a combination of 3D structural and residue interaction network analyses to support roles for residues 21, 62, 104, 112–117, and 122 in lysozyme amyloidosis. By comparing two amyloidogenic variants to WT lysozyme, we were able to identify network and 3D structural changes that were shared between the amyloidogenic variants. Modeling dimer and trimer interactions further supported a role for these residues. These residues appear to be especially important for trimer formation. This study generates several hypotheses that can be experimentally tested. 1) Residues 112–117 are less flexible in amyloidogenic variants of lysozyme than in the WT. 2) Residues 21, 62, 104, 112–117, and 122 are involved in the primary nucleation of lysozyme by facilitating intermolecular interactions between mutant lysozyme molecules. 3) Trimers of lysozyme are more stable than dimers. 4) Lysozyme mutant molecules favor self-interactions over interactions with WT molecules. 5) Interprotein interactions in or close to the native state likely play a larger role in amyloid formation in general than previously hypothesized. This study demonstrates the utility of combining 3D structural and network analysis for understanding amyloid formation. Furthermore, it provides insight into lysozyme amyloid formation that may be applicable to the study of many other amyloidoses.

## Abbreviations

OMIM: Online mendelian inheritance in man
PDB: Protein data bank
PGR: Protein graph repository
RIN: Residue interaction network
RING: Residue interaction network generator
RMSD: Root mean square deviation
WT: Wild type
